# Methodological Approaches to Study Extracellular Vesicle miRNAs in Epstein–Barr Virus-Associated Cancers

**DOI:** 10.3390/ijms19092810

**Published:** 2018-09-18

**Authors:** Li Sun, David G. Meckes

**Affiliations:** Department of Biomedical Sciences, Florida State University College of Medicine, Tallahassee, FL 32306, USA; li.sun@med.fsu.edu

**Keywords:** exosomes, oncosomes, microvesicles, microRNA, extracellular vesicle, herpesvirus

## Abstract

Epstein Barr-virus (EBV) was the first virus identified to be associated with human cancer in 1964 and is found ubiquitously throughout the world’s population. It is now established that EBV contributes to the development and progression of multiple human cancers of both lymphoid and epithelial cell origins. EBV encoded miRNAs play an important role in tumor proliferation, angiogenesis, immune escape, tissue invasion, and metastasis. Recently, EBV miRNAs have been found to be released from infected cancer cells in extracellular vesicles (EVs) and regulate gene expression in neighboring uninfected cells present in the tumor microenvironment and possibly at distal sites. As EVs are abundant in many biological fluids, the viral and cellular miRNAs present within EBV-modified EVs may serve as noninvasion markers for cancer diagnosis and prognosis. In this review, we discuss recent advances in EV isolation and miRNA detection, and provide a complete workflow for EV purification from plasma and deep-sequencing for biomarker discovery.

## 1. Introduction

MicroRNAs (miRNAs) are small noncoding RNAs with a length of 20 to 25 nucleic acid base-pairs. miRNAs are key regulators of gene expression mainly through mRNA degradation or translation inhibition. In addition to the profound effects of miRNAs within cells, miRNAs are also released into the extracellular space in complex with proteins or encased within extracellular vesicles (EVs) [1]. EVs are a diverse collection of membrane-bound sacs that typically range in size from 40 to 500 nm and are further classified based on the site of subcellular formation [2]. Exosomes are small EVs ranging in size from 30 to 150 nm in size that are produced at internal, endosomal-derived membranes of the multivesicular bodies. Microvesicles are generally larger EVs greater than 100 nm in size and formed following budding and fusion events at the plasma membrane [3]. With current methods and technology, it is nearly impossible to separate the two types, as the vesicle populations have overlapping sizes, densities, and contain similar markers [4]. For this reason, we will use the term EVs when describing results presented throughout the literature. One of the most important biological properties of EVs regardless of the subcellular origin is their ability to transfer specific profiles of proteins, lipids, RNAs, and even DNA fragments between cells to mediate intracellular communication events [1,5,6].

Cell-to-cell communication within the tumor microenvironment plays a critical role in cancer development and progression. Cancer cells must communicate with surrounding cells in order to proliferate, induce angiogenesis, evade the immune system, invade surrounding tissue, and metastasize to other sites in the body [7]. Many of these processes are regulated by miRNAs within cancer cells [8,9,10,11,12]. Moreover, miRNAs are also released from primary tumor tissues into the bloodstream and can be found in a wide variety of biological fluids [13]. Thus, miRNAs represent a new class of circulating cancer biomarkers that participate in EV communication events [14,15,16]. It is clear from many studies that miRNAs have specialized functions and play important roles in inter- and intracellular signaling [17,18]. Therefore, the study of miRNAs in cancer is likely to shed light on the mechanisms driving cancer progression and result in novel biomarkers for disease. In general, the correlation between miRNAs and a specific type of cancer is based on the up- or down-regulation of miRNA and miRNA expression patterns. For example, one of the most studied miRNAs, miR-21, is found in human serum samples from patients with different solid tumors (breast, colon, and lung) and promotes tumor growth [11,19].

Traditionally, cancer detection and diagnosis are obtained following a conventional tissue biopsy, which involves the removal of small portion of tissues from the primary tumor growth site via a surgical resection followed by histopathological examination and cancer staging. In some cancers, specific tissue biomarkers have been described that may result in earlier diagnosis, staging, and patient survivability. Compared with conventional diagnostic methods, the use of EVs as biomarkers has several advantages. First, EVs are abundant in nearly every biological fluid analyzed. For example, there are more than 10^12^ EVs in 1 mL of blood compared to fewer than 10 circulating tumor cells (CTCs) [20,21,22]. Second, EVs contain specific molecular profiles representing the cell of origin and have been found to participate in many aspects of cancer development and progression [23,24,25,26,27]. Also, a recent paper demonstrating that the miRNA profiles are similar in plasma and EVs for healthy samples, but significantly higher in EVs than plasma from lung cancer patients [28]. These data provide evidence that EVs are enriched in tumor biomarkers. Third, miRNAs and other molecules are well protected by a lipid membrane and more stable under RNase treatment or other storage conditions [29]. These properties make the detection results more reliable. EVs have become one of the ideal candidates for non- or minimally-invasive liquid biopsies for multiple human disease states.

## 2. EBV miRNAs in EBV-Associated Cancers

Epstein–Barr virus (EBV) was the first virus identified to be associated with human cancer. In 1964, Epstein and colleagues discovered a virus in suspended cultured African Burkitt’s lymphoma (BL) cells using electron microscopy [30]. EBV produces a latent and persistent infection within circulating lymphocytes and is found ubiquitously throughout the world’s population [31,32]. Although most infections remain benign and asymptomatic, EBV is also associated with multiple human cancers of both lymphoid and epithelial cell origins, including lymphomas (BL, Hodgkin’s lymphoma (HL), and post-transplant) and carcinomas (gastric and nasopharyngeal) [31]. The prognosis and survivability of human cancers caused by EBV and other DNA viruses that encode miRNAs may greatly benefit for the development of EV-based diagnostic platforms.

According to the Sanger miRBase (Release: 22 March 2018), a total of 25 EBV miRNA precursors with 44 mature miRNAs are mapped to the Bam HI fragment H rightward open reading frame 1 (BHRF1, four miRNAs) and Bam HI A rightward transcripts (BART, 40 miRNAs) regions of the EBV genome [33]. The BHRF1 cluster is from a transcript that encodes the BHRF1 protein. However, the BART miRNAs are transcribed from a large transcript (about 22 kb) which has two alternative promoters [34]. An early paper reported only two BART miRNAs because the B95.8 strain of EBV has an 11 kb deletion in the BART transcript [35]. The missing region includes all of BART cluster 2 (13 miRNAs) and part of cluster 1 (four miRNAs). Therefore, it is evident that the BART miRNAs in the deleted region are not required for EBV immortalization of resting B cells in vitro.

EBV encoded miRNAs have been implicated in regulating both viral and host targets for inhibiting apoptosis, promoting cell growth, and controlling latent EBV infection [36,37,38,39,40,41,42,43,44,45,46]. Using ΔmiR EBV strains, Tagawa et al. found secretion of IL-12 from infected B cell is significantly increased compared with wt/B95-8 virus [47]. Further experiments show that EBV BART1, BART2, and BHRF1-2 target the IL12B gene to prevent Th1 differentiation of naive CD4+ T cells. Moreover, these viral miRNAs could also affect antigen presenting processes by targeting molecular MHC I/II, lysosomal enzymes, and the transport protein TAP1/2 of antigenic peptides into the ER lumen [47,48]. Viral proteins like EBNA1 [48] and latent membrane proteins like LMP1 [40] or LMP2A [42] could also be downregulated by EBV miRNAs to escape immune surveillance and maintain persistent infection. BART2 was found to bind to the 3′UTR of EBV DNA polymerase BALF5, which could maintain EBV latency [35,41]. Additional evidence supports the hypothesis that EBV miRNAs help infected cells to survive by inhibiting apoptosis and increasing cell cycle progression and proliferation [36,49,50,51].

### 2.1. Nasopharyngeal Carcinoma (NPC)

Analysis of EBV positive cell lines and clinical NPC tissues revealed that only lytic infection or cells with type III latency expression profiles express BHRF miRNAs. Four independent research groups showed that no miRNAs are expressed from BHRF1 cluster in NPC samples, which is consistent with a type II latency expression profile [33,38,52,53]. However, all other EBV-infected cell lines with other latency patterns have a detectable level of all BART cluster miRNAs [52,54]. Among these expressed miRNAs, BART7 always shows the highest expression level regardless of cell or latency type [54].

Two different groups have used deep sequencing technology to characterize the EBV miRNA transcriptome in NPC tissues [33,53]. Zhu et al. found EBV miRNAs make-up 3.8% and 18.2% of total miRNAs in NPC tissues [53]. Chen et al. identified all 44 EBV BART miRNAs, including four new mature miRNAs derived from previously identified BART miRNA precursor hairpins [33]. About 23.2% of the total miRNAs were EBV miRNAs in their samples. Several EBV miRNAs were expressed, some at levels similar to highly abundant human miRNAs.

The main challenge in the successful treatment of nasopharyngeal carcinoma is the difficulty of early detection and accurate prognosis of the disease. A large proportion of NPC patients (∼70%) are diagnosed at later stages of the disease [55,56,57]. Once metastasis occurs, the disease progresses rapidly with poor clinical outcomes. This is partly a result of inadequate understanding of molecular and cellular pathogenesis of NPC. In addition, we lack biomarkers for effective early diagnosis and patients exhibit a modest response to current therapies. The expression profiles of miRNAs in the circulation may represent a reliable biomarker for NPC diagnostic and prognostic purposes. Moreover, miRNAs have even shown therapeutic potentials in some kind of cancers [58,59,60].

EBV miRNAs have been proposed to serve as diagnostic markers in patients with NPC [61], for the level of serum miRNAs is positively correlated with the copy numbers of host miRNAs in tumor biopsies [38,62]. For example, Liu et al. reported five plasma miRNAs that in combination (upregulation of miR-16, miR-21, miR-24, and miR-155 and downregulation of miR-378) could be used as a diagnostic standard for NPC, providing 87.7% sensitivity and 82.0% specificity [63].

In addition to NPC, other cancers caused by EBV would benefit from the ability to be quickly diagnosed from a minimally invasive procedure. Below details what is currently known about the viral miRNA expression patterns in other EBV-associated cancers.

### 2.2. Diffuse Large B-Cell Lymphoma (DLBCL) and Natural Killer/T-Cell Lymphoma (NKTL)

Imig and colleagues compared EBV-positive versus EBV-negative DLBCL clinical samples by deep-sequencing to profile miRNAs expressed by EBV and host cells [64]. The virus-encoded miRNAs represented approximately 2% of the overall miRNA count and all known EBV miRNAs with the exception of the BHRF1 cluster as well as BART15 and BART20 were present. The highest expression was found for BART7, BART22, and BART10. A similar approach was used later on NKTL samples [65] by the same group. All BART miRNAs were detected and about 2.2% total miRNAs were from EBV.

### 2.3. BL and Lymphoblastoid Cells (LCL)

Many studies have described EBV miRNAs expression profile in lymphocytes using EBV-infected cell lines. In 2004, Pfeffer et al. [35] found five EBV encoded miRNAs in B95.8-transformed cells. Later in 2006, Cai et al. found BHRF miRNAs express only in latency III, but BART miRNAs are expressed at high levels in latently infected epithelial cells and at lower levels in B cells [66]. At the same time in 2006, 18 miRNAs were first predicted by computational method and then confirmed by microarray [67].

### 2.4. Gastric Carcinoma (GC)

BART1, 3, 5, 7, 10, and 12 were detected by northern blot analyses in an EBV-infected gastric cancer cell line and animal model sample of the disease [44]. Marquitz et al. demonstrated that an EBV-infected GC cell line shows limited viral protein expression, whereas BART miRNAs are abundantly expressed and produced transformed phenotypes when compared to noninfected GC cells [68]. The first comprehensive study of all 44 EBV miRNAs in GC clinical samples was finished by Aya Shinozaki-Ushiku et al. on 2015 [69]. As seen in NPC and other EBV related cancer samples, BART7 shows the highest expression level [54,64]. They also proved that BART4-5p inhibit apoptosis by downregulating Bid protein. Another systemic profiling of EBV miRNAs expressed in GC patients was done by Tsai and colleagues in 2017 [70]. The most abundant EBV miRNAs of GC were BART4, followed by BART11, BART2, BART6, BART9, and BART18. Kim et al. isolated and characterized a new EBV-infected GC cell line (YCCEL1) that was established from a Korean patient with EBV-associated GC [71]. YCCEL1 cells expressed BART miRNAs at high level but did not express BHRF1 miRNAs, which is consistent with other data from GC samples and cell lines.

### 2.5. Comprehensive Studies

Two comprehensive studies comparing EBV miRNA expression profiles in distinct cell lines were published in 2011 [72,73]. Qiu et al. compared a variety of EBV related tumor and neoplasias by using clinical biopsies, primary cell, and established cell lines [72]. They found that miRNAs have distinct expression levels in EBV-related epithelial cancers when compared with lymphoid malignancies. Their data also provides evidence that there are specific expression patterns of viral miRNAs for each latency program. BHRF1 cluster miRNAs are only expressed in latency III but not in type I and type II latency. These patterns are disrupted in EBV associated tumors, implicating EBV miRNAs in viral persistence and oncogenesis. EBV miRNA expression patterns could be used to distinguish EBV tumor types, but there was no subset of miRNAs that was uniquely responsible for discriminating the types of tumors.

Another study by Amoroso et al. also used a massive collection of EBV-infected cell lines with different latency types and tumor origins to study miRNAs expression profiling [73]. During the latency phase, BART miRNAs could be detected in all forms of infection but expression level of different miRNAs may vary up to 50-fold. BHRF1 miRNAs were only seen in cells with detectable Cp- and/or Wp initiated EBNA transcripts. When entering into lytic phase, BHRF1-2 and BHRF1-3 were expressed within BHRF transcripts while BART miRNAs level remains stable.

Taken together, the EBV miRNA expression profiles are complex and tumor- and latency-type specific. EBV encoded miRNAs expression in the various cancers are summarized in Table 1.

## 3. EBV miRNAs and EV

In addition to regulating host gene expression through viral-encoded miRNAs, EBV infection also alters the protein cargo of EVs released from infected cells [74,75]. However, molecular cargo variations in EVs induced by the virus are not limited to protein and also include nucleic acids such as viral mRNAs [76] and miRNAs [77,78]. EBV-modified EVs containing BART miRNAs were first reported to be released continuously from LCL [76]. Shortly thereafter, these BART miRNAs containing EVs were confirmed to be released by infected NPC cells (C666-1) by our group [79] and the Busson lab [78]. Moreover, BART1-5p, 5, 7-3p, 12, and 13 have also been found in the circulating EVs from patients with NPC [78]. Our group also detected miRNAs and the viral oncoprotein LMP1 in EVs present in the serum of mice carrying NPC tumor xenografts and others have found these molecules in the serum of NPC patients. The transfer of EVs containing LMP1 was found to activate signal transduction pathways and produce phenotypic changes in recipient cells [79,80,81,82].

A study by Pegtel et al. provided the first evidence for functional miRNA deliver via EVs [76]. Specifically, EBV-infected B cells secrete BHRF1-3 in EVs that can reduce target gene expression in uninfected recipient cells. Furthermore, they also detected EBV-encoded BART cluster 1 miRNAs in circulating non-B cell lymphocytes derived from EBV-infected patients, where EBV DNA is not present. The data suggested that the presence of miR-BARTs in these cells can be attributed to EV transfer.

It is thought that EBV miRNAs in EVs play important roles in controlling the innate and adaptive antiviral immune responses. After being secreted by EBV-infected cells, EVs can be taken up by different cell types, including monocytes and monocyte-derived dendritic cells [76], plasmacytoid dendritic cells [83], T cells [84], epithelial cells, endothelial cells, and fibroblasts [81]. Transfer of viral miRNAs to cells can lead to the repression of target genes [76]; for example, BART15 represses the inflammasome protein NLRP3 in a monocytic cell line [85,86]. Rechavi et al. reported that EBV miRNAs can be transferred from infected B cells to non-EBV-infected T cells and silence the target gene expression in the recipient T cells [84]. BART miRNAs have a wide range of targets, including PTEN (PI3K/AKT pathway), Wnt pathway, and the tumor suppressor genes WIF1, NKD, CXXC4, and APC [38]. Therefore, it is likely that BART miRNAs in EVs may target similar pathways when delivered to noninfected cells. Future investigation into the levels of these miRNAs in EVs purified from patient samples may lead to better diagnostic or prognostic markers.

## 4. EV Isolation Methods

There are many methods to isolate EVs from biological fluids to study their functions, for biomarker discovery and liquid biopsy development. From the most classic ultracentrifugation method to well-developed commercial kits, the ultimate goal is to isolate EVs from complex biofluids with high purity and recovery. With all available approaches, it is critical to remove non-EV cellular debris and proteins which may contaminate the downstream miRNA detection. For plasma or serum samples, it is important to defibrinate and avoid hemolysis. Fibrin-clotting will reduce the EV purity and clog membranes or columns used in some EV isolation methods. Additional caution must be taken to prevent the release of miRNAs from red blood cells during hemolysis and ensure the accuracy of exosomal miRNA detection [87]. Therefore, samples must be processed rapidly with an appropriate anticoagulant. For example, heparin is not a suitable anticoagulant for EV isolation because it may inhibit downstream enzymatic reactions. EDTA is an anticoagulant of choice when processing blood for EV isolation and miRNA detection. However, more detailed studies on the effects of anticoagulants on EVs and downstream analyses is needed [88]. Following blood processing, EVs can be extracted using numerous methods with associated advantages and disadvantages.

### 4.1. Ultracentrifugation (UC)

Differential ultracentrifugation (DC) was the first EV purification method described in the literature and has long been considered the gold standard for the isolation of relatively homogenous size populations of small EVs. DC is a series of centrifugation steps with increasing centrifugal force to enrich EVs and remove unwanted cellular materials. This method is often used in combination with sucrose or iodixanol density gradients to purify EVs from contaminating protein complexes and separate EV populations based on size and density. One limitation of density-gradient UC is that this approach cannot purify EVs from contaminants with overlapping densities. For example, the density of HDL considerably overlaps with vesicles and frequently copurifies with EVs [89,90]. In addition to these technical challenges, the method is very labor-intensive, time-consuming, and dependent on highly-trained researchers and expensive instrumentation. Therefore, UC is not an ideal method for rapid EV isolation in a clinical setting for diagnostic purposes. Despite the limitations of DC, it continues to be an important method for the study of EV biology and the standard with which new methods are measured against.

### 4.2. Ultrafiltration (UF)

EVs can be purified from smaller contaminating material by UF based on filter poor size. Typically, filters with molecular weight cut-offs of 100 kDa are used to concentrate small EVs greater than or equal to 30 nm in size and remove soluble proteins [91]. However, this method also retains a large quantity of albumin and immunoglobulin on the membrane that significantly reduces the purity of EV preparations. It is for this reason that UF is frequently combined with other methods to obtain more pure isolates [92,93]. A major advantage of UF is the ability to concentrate large quantities EVs from fluids that maintain biological activity. Heinemann et al. developed a three-step protocol to isolate large volumes of biofluid based on sequential steps of dead-end prefiltration, tangential flow filtration (TFF), and low-pressure track-etched membrane filtration [94]. This strategy is scalable and could potentially be developed into a fully automated system.

Small UF devices (e.g., vivaspin, Amicon, Pellicon, and Minimate) can be operated using standard centrifuges or mechanical pumps. Therefore, UF could be easily employed in most diagnostic laboratories and commercial kits are now available to take advantage of this method of EV enrichment. For example, the ExoMir kit from Bioo Scientific uses positive pressure to drive fluid sample though tandem microfilters to remove cellular debris and capture all vesicles of diameter larger than 30 nm.

### 4.3. Size-Exclusion Chromatography (SEC)

SEC or gel-filtration chromatography is a chromatographic method in which molecules in solution are separated by their size similar to UF. SEC works through the principle that smaller objects take longer time to travel through the matrix. Therefore, SEC is an ideal method to separate EVs from soluble proteins, and larger protein aggregates like albumin and Ig [93].

SEC has several advantages when compared with other methods:

First, SEC does not require the addition of other reagents and always maintains the samples in physiological buffer conditions, which is important to maintain the biological properties of EVs. Compared to differential centrifugation, there is little risk of vesicle aggregation and copurification of soluble proteins and protein complexes. The plasma sample needs to be diluted in UC due to its high viscosity, otherwise the recovery efficiency of vesicles is greatly reduced. Sucrose is frequently used for density-gradient purification of vesicles and membrane proteins, but this method of isolation has been shown to affect vesicle size and their biological properties [95].

Secondly, SEC does not require expensive instrumentation and is less labor intensive than UC. Many groups have now shown the separation of serum EVs in as little as 10 min on columns packed with sepharose matrix [96,97]. Other methods require longer incubation and centrifugation times to isolate EVs from liquids. EV samples can be prepared for analysis on the same day of collection which is particularly advantageous in a clinical setting. In addition, sepharose CL-2B SEC packed columns are relatively inexpensive and are reusable [96]. For researches that do not want to prepare their own columns, traditional commercially available SEC columns [93] specially designed for EV isolation exist, like qEV [22,98] from Izon, for an added cost.

Despite the advantage of SEC, several disadvantages exist. For example, unlike other methods, SEC is the only method which does not concentrate samples, but instead dilutes them. So, SEC requires additional steps to enrich EVs for downstream applications, which results in lower overall total recovery. Isolation of vesicles with sepharose CL-2B SEC leads to 30–70% vesicle recovery before concentration, compared to 80% with UC [96]. Another issue with SEC is that it cannot handle large volumes of sample. Therefore, a concentration step like UF must be applied before SEC when working with large quantities of biological fluids [92,93].

### 4.4. Anion Exchange Chromatography (AIEX)

EVs have been found to have a net negative charge similar to the plasma membrane of cells. Recently, researchers have started to take advantage of this property to purify EVs by AIEX. When compared to UC and TFF, AIEX was found to be comparable in yield to UC with decreased protein and debris contamination compared to EVs isolated by TFF [99]. The high flow rates possible with AIEX allow for the purification of 10^11^ EVs from 1 L of cell culture supernatant in less than 3 h. The rapid and scalable purification of EVs by AIEX will aid in the development of clinical application of EVs including drug delivery and biomarker discovery.

### 4.5. Precipitation Methods

Volume-excluding polymers such as polyethylene glycols (PEGs) are routinely used for precipitation of viruses, bacteriophage, and other small particles including EVs [100,101,102]. The precipitate can be isolated using either low-speed centrifugation or filtration [103]. Again, like most methods, commercial products are available. Total EV Isolation reagent (TEI, invitrogen) and ExoQuick (SBI) are two commercial kits that take advantage of PEG-based precipitation of EVs. These kits have optimized protocols for the isolation of EVs from cell-culture media, serum, plasma, urine, and other body fluids (saliva, milk, cerebrospinal fluid, ascites fluid, and amniotic fluid). Many groups have shown that the precipitate-based method induce large protein complexes and EV aggregation, which reduce the purity compared with other methods [22,104,105,106]. However, we have demonstrated that this method is very efficient with recovery rates greater than 90% [107].

### 4.6. Immunoaffinity Isolation

To isolate specific EV-subpopulations, vesicles can be affinity purified with antibodies to EV surface markers like tetraspanins (CD63, CD81, and CD9), annexins, EpCAM, integrins, EGFR, and MHC. Antibodies directed against these surface proteins can be used individually or in combination. For this application, the antibodies are immobilized on a variety of media, including magnetic beads, chromatography matrices, plates, and microfluidic devices [89,108,109,110]. Researchers using this method should be cautious in interpreting data, as there are many EV subtypes with different surface protein expression profiles [111,112]. It is also likely that the EV surface protein profiles differ between cell types and cellular conditions [113]. Regardless of these limitations, affinity-based purification is useful in distinguishing subpopulations and isolating EVs from different organs and tissues, especially under physiological conditions. Additionally, it can be combined with antibody-based methods to merge purification and downstream analyses [114].

Commercial kits exist, like EV isolation kit from MACS, for affinity-based EV isolation. MACS provides magnetic beads with specificity to tetraspanins CD9, CD63, and CD81. They also offer a MACSPlex EV kit for surface protein profiling. Qiagen has developed a well characterized kit named exoEasy or exoRNeasy Serum/Plasma which utilize a spin column and affinity membrane-based method to isolate EVs. When combined with the company’s miReasy kit, total EV RNAs can be quickly purified for molecular characterization [115].

Other affinity-based EV purification techniques have been described that utilize the surface properties of EVs. For example, EVs are known to have phosphatidylserine (PS) displayed on the surface. Nakai et al. used the PS binding protein TIM4 to isolate highly purified EVs [116]. The EVs isolated using this method could be eluted with the addition of chelators as the binding of TIM4 to PS is Ca^2+^-dependent. Another approach takes advantage of the affinity of EVs for heparin [117]. Heparin coated beads or columns can be used to enrich EVs from biological fluids. One limitation of this approach is that other contaminating biomolecules also have an affinity for heparin including some antibodies, coagulation factors, DNA-binding proteins, lipoproteins, and other plasma proteins [118,119,120,121,122].

### 4.7. Microfluidic Devices

Microfluidic technology allows one to control fluids on a small, typically submillimeter, scale which is ideal for the development of EV diagnostic tests. Usually the device is a set of microchannels etched or molded into a solid material (like glass or silicon) in a chemistry or engineering lab. Currently, this technology is not widely used in traditional biomedical research labs, but the potential still remains. Microfluidic chips which capture EVs by affinity have been discussed above, here we introduce two types of microfluidic-based EVs isolation methods: acoustic nanofiltration and viscoelastic flow sorting.

The separation of EVs by acoustics is based on ultrasound standing waves that exert differential acoustic force on EVs according to their size and density. Lee et al. optimized the ultrasound transducer to achieve a 200 nm size cut-off and ~70% recovery on cell medium sample [123]. Another recently implemented microfluidic approach for sorting of EVs is viscoelastic microfluidics, which use elastic force to separate particles based on size. To control the viscoelastic force of media, Liu et al. diluted EV samples in poly(oxyethylene) (PEO) solution and were able to achieve similar purity and recovery as previous methods routinely used in the field [124].

The extremely low processing capacity and long operation times greatly restricts the use of microfluidics in general lab applications. Furthermore, these methods need more validation and standardization. However, once a biomarker is established for diagnostic purposes, microfluidics may prove to be an ideal method for detection small volumes of clinical samples.

### 4.8. Combination Approaches

Considering there are pros and cons for each single method (see Table 2), researches have started to combine and further optimize the methods discussed above to improve recovery and purity. Unfortunately, the additional steps usually required to increase purity often result in a reduction in yield. Therefore, the downstream application must be taken into consideration when choosing the method of EV isolation.

UC and UF are often used to concentrate large volumes of biological fluids prior to SEC separation [92]. Koh et al. described an approach of preprocessing of plasma by UC followed by SEC to isolate and enrich EVs. This method provided the best yield as determined by nanoparticle tracking analyses and the presence of the exosomal markers CD63, Flotillin-1, and TSG-101. EV morphology was verified by transmission electron microscopy and found to remain intact [98]. Concentration of EVs using precipitation can also be combined with SEC. However, depending on the source material these approaches will affect recovery and potentially increase protein contaminants, negatively impacting vesicle purity [97].

PEG isolated EVs were found to have similar purity to UC if a small volume PBS wash step is included in the EV isolation work-flow. Our group has optimized a PEG-based precipitation method termed ExtraPEG which first enriches EVs from large quantities of media with PEG, followed a PBS wash ultracentrifuge step to improve purity. The quality of proteins and RNAs from ExtraPEG method is sufficient for proteomics and deep-sequencing [107]. Furthermore, highly pure EVs were obtained when PEG was combined with density gradient UC methods [104]. More recently, we have found that this method of EV enrichment can be combined with other methods like SEC and affinity-based purification.

Exo-spin is a commercially available kit that combines precipitation with column-based purification. After EVs are concentrated by precipitation, the EVs are further purified on a small spin column.

### 4.9. Comparison of EV Isolation Methods for miRNA Detection

Recent evidence suggests that the EV isolation method can introduce bias in the miRNAs detected [105,125,126,127]. When comparing UF and precipitation-based methods, precipitation offers higher EV recovery and some bias for specific miRNAs. Rekker and colleagues found a strong correlation of EV miRNA profiles that was dependent on the EV isolation method [125]. Specifically, miR-92a and miR-486-5p were significantly influenced by the EV purification method chosen prior to analysis. Schageman and colleagues [128] compared UF with TEI and demonstrate that both protocols isolate relatively pure EV populations, but the TEI reagent consistently recovered more EVs as determined by a 1–3 Ct shift on quantitative polymerase chain reaction (qPCR) for different RNAs. When using next-generation sequencing (NGS) techniques to examine EV RNA content, both methods showed the same trend on the five miRNAs tested. Unfortunately, the NGS kit used in this study was not designed for small RNAs, so less than 10% of total reads were miRNAs. Another group compared the Exoeasy kit to SEC [129]. They found that EVs isolated by exoEasy kit have a bigger median diameter, larger size range, and a greater yield when compare with SEC qEV column. However, ExoEasy recovered more low-density lipoprotein contamination but total RNA yield was comparable and in the 3 to 7 ng per mL of plasma range.

A more comprehensive comparison has been carried out by some other groups. Andreu et al. tested PEG-based precipitation with other methods based on UC, columns or filter systems [130]. They concluded that the overall performance of PEG was very similar, or better than other commercial precipitating reagents, in both protein and miRNA yield. However, Lobb et al. [22] compared similar methods and found that current precipitation protocols for the isolation of EVs from cell culture conditioned media and plasma provide the least pure preparations of EVs. According to the authors, SEC produced results similar to density gradient purification of EVs with high particle purity. They suggest combing UF with SEC to isolate highly pure EVs from cell culture medium and human plasma in an efficient time frame. Tang et al. not only compared different isolation methods but also various RNA extraction kits [105]. Their results similarly demonstrated that precipitation methods (ExoQuick and TEI) result in higher isolation efficiency than traditional UC, but also have higher protein contamination. More total RNAs were isolated from precipitation methods, but RNA chip analysis shows that some of this may be from the protein contamination. Further RNA sequencing analyses proved that the isolation method can influence the small RNA profile. Recently, Buschmann and colleagues used NGS to compare different EV isolation and RNA extraction methods. Their results show that each method generates biases on EV subpopulations as well as contaminations [127]. While precipitation and membrane affinity methods give the highest miRNA reads, SEC based methods provides the greatest purity EVs isolates.

Methods of RNA extraction from EVs has also been compared by Eldh et al. [126]. For all methods evaluated, RNA of sufficient quality and purity was obtained as determined by RNA integrity number (RIN) and OD values. In their hands, a column-binding approach resulted in the highest RNA yield and the broadest RNA size distribution. Other phenol-based methods resulted in lower total RNA recover that was enriched in small RNAs including miRNAs.

In addition to cell culture media and plasma, the EVs isolation efficiency from urine samples has been evaluated by different groups. Wachalska et al. [131] demonstrated that SEC could separate EVs from the protein-complex fraction (THP-protein-network), whereas the other two commercial kits could not. These precipitation-based kits resulted in lower miRNA recovery from the reverse transcription qPCR (RT-qPCR) test of three miRNAs (miR-375, miR-204, and miR-21). They also found that these three miRNAs have a different ratio in EV fraction and protein fraction, which proved the importance of EV purity. Royo et al. compared five different methods based on UC, precipitation and lectin binding [132]. RNA profiling results with miRNA arrays showed high correlation between all tested methods and kits. Channavajjhala et al. [133] found that UF in combination with SeraMir exoRNA columns represents the optimal miRNA purification procedure from urinary EVs. Moreover, storage conditions of urine do not influence the relative abundance of urinary exosomal miRNAs.

**Figure 1:**
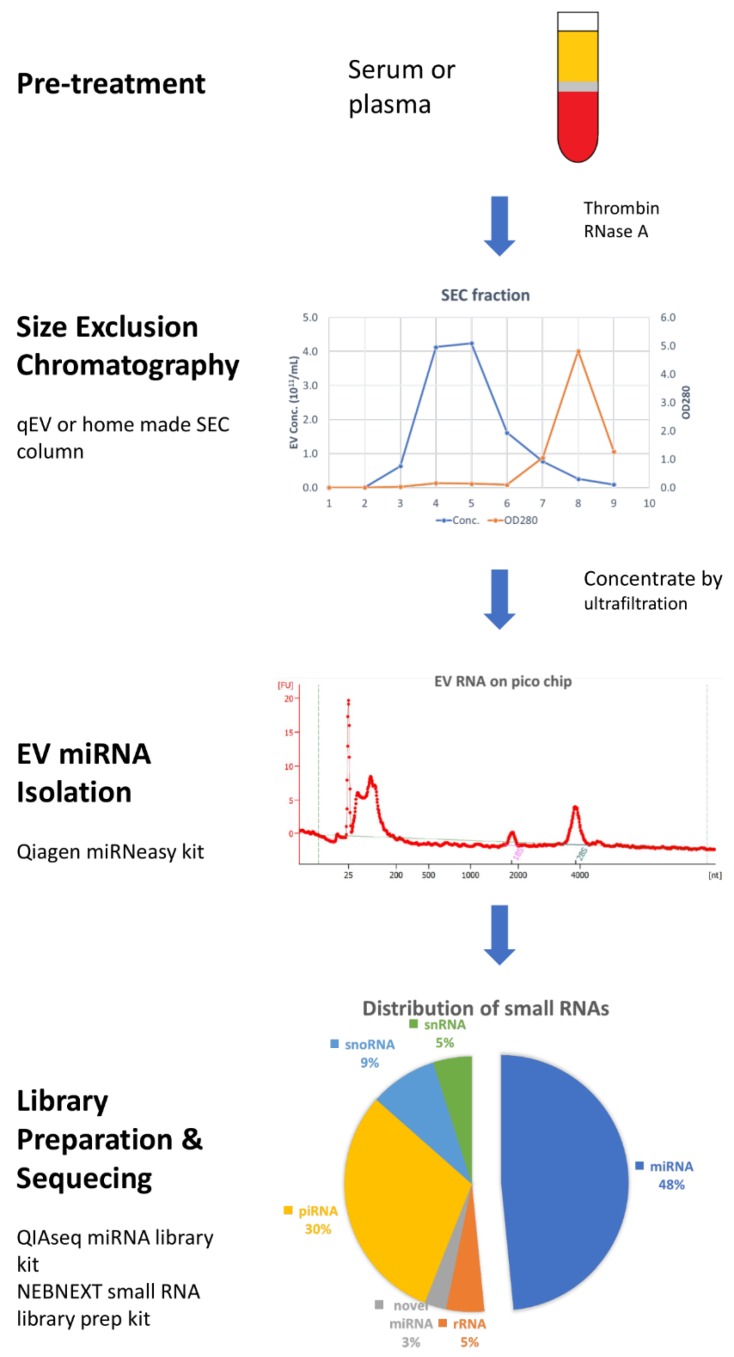
Workflow of miRNAs profiling from blood extracellular vesicles (EVs). Plasma or serum samples were pretreated with thrombin and RNase A to remove fibrin and non-EV RNA contaminate. 1 mL of cleared plasma was loaded on size-exclusion chromatography (SEC) column and 1 mL fraction from 3 to 6 were collected and pooled. Concentrated EVs samples were quantified by nanoparticle tracking analysis (NTA), and 50% to 80% recovery could be achieved. Total EV RNAs were isolated by Qiagen miRNeasy micro kit. After checking total RNA with pico chip on a bioanalyzer, small RNA libraries were prepared by using NEBNext Small RNA Library Prep Set and run on an Illumina sequencer.

Taking all these results together, most researchers agree that the SEC method produces purer EVs than precipitation and UC. Based on the recent findings and developments in EV isolation and RNA characterization, we provide a complete work-flow for the analysis of EV miRNA cargo from plasma samples (Figure 1) that can easily be adapted to other types of biological fluids or RNA and DNA species.

## 5. Methods for miRNA Detection

EV samples of sufficient quantity and purity are important for downstream applications. Total RNA should be prepared using methods that preserve small RNA species. Once the EV RNA is extracted, there is no technical difference between handling miRNA from cells and EVs. Generally, the levels of EV RNA will be low and in the nanogram range. However, RNA yield is dependent on the number of EVs isolated and can be scaled-up accordingly. After RNA purification, there are several established techniques for miRNA detection, such as northern blotting, RT-qPCR, microarray, NGS, highly sensitive biosensors (including molecular beacons), and electrochemical-based methods (like surface plasmon resonance) [134].

The first method developed for miRNA detection was Northern blotting, which was used in the initially discovery of lin-4 miRNA [135]. Similar to Western blotting, this technique separates biomolecules by electrophoresis and then transfers miRNAs to a positively charged nylon membrane. Finally, labeled probes are hybridized with the miRNAs immobilized on the membrane and imaged. Our group has successfully used this approach to detect EBV miRNAs in EVs isolated from latently infected cancer cells [79]. Northern blotting is specific and semiquantitative; however, it is time consuming approach. Other techniques now exist for the rapid detection and analysis of miRNAs.

### 5.1. RT-qPCR

RT-qPCR has been wildly considered as the go to standard for miRNA detection. It provides excellent reproductivity, sensitivity, and dynamic range at a reasonable cost. Under optimal conditions, qPCR can provide great linearity with total RNA input from 1 pg to 1 μg and successful detection as low as 10 copies of cDNA in a sample. However, this method can become labor intensive and expensive when used in high throughput screening or for whole transcriptional profiling. For these reasons, it is frequently used to validate results from other miRNA profiling methods, like NGS and microarray. One advantage of PCR-based methods is the ease at which they can be adapted for clinical use. Indeed, many diagnostic tests take advantage of this technology and are already used in the clinic.

The first step in miRNA detection is to transform the miRNAs into cDNA by reverse transcription. As there is no poly-A tail at the 3-end, reverse transcription needs to be carried out by either using miR-specific primer or using universal primer after adding poly-A by polymerase.

The miR-specific primers have a stem-loop structure at their 5-end and a miRNA antisense portion of approximately 6–8 nucleotides on their 3-end [136]. The 5-end in the stem-loop also contains a designed nucleotide sequence for both reverse transcription and qPCR. Pre-miRNA exists as a stable hairpin of approximately 70 nts in length which make it impossible for stem-loop primer to hybrid at 3-end of unmatured miRNAs [136]. This feature greatly enhanced specify and reduced background, but somehow limit its use in profiling approach [137].

The most widely used approach is to convert miRNAs into poly-A tailed RNA species is with poly-A polymerase (PAP) [138], which is the fundamental principal of many commercial products. On the second step, an RT primer which contains both poly-T and universal qPCR segment binds to the extended miRNAs for cDNA synthesis. The disadvantage of this method is that PAP extends all RNAs including pri-/pre-miRNAs as well as other small RNAs which may reduce specify of qPCR detection. Moreover, the predesigned RT primer highly restricts Tm optimization in qPCR. miQPCR is another similar approach where a universal RT primer is linked to 3-end of miRNA by T4 RNA ligase prior to utilizing the same cDNA synthesis step [139].

Another approach is dependent on designed stem-loop primers which hybridized on the 3-end and/or 5-end of target miRNA [140,141,142]. Li and Zhang published similar two-primers approaches in 2009 and 2011, respectively [140,141]. After hybridized with the half sequence of the target miRNA, the two primers are immediately adjacent to each other which can be ligated by the catalysis of T4 RNA ligase and used as template in the next qPCR step. Later in 2017, Androivic et al. improved this approach by using a hair-pin primer composed of two hemi-probes complementary to each end of target miRNA [142]. This dual binding approach greatly increase the sensitivity and specificity of qPCR detection on miRNAs.

Following cDNA synthesis by any of the methods mentioned above, qPCR can be performed as in mRNA detection by using miR-specific primers or a universal primer set. The two fluorescent systems available to monitor qPCR reaction are SYBR Green and TaqMan probe-based methods. For either approach, internal tests should be performed to determine amplification efficiency and specificity. Usually, a 10-fold dilution assay could test for the existence of any PCR inhibitor, and amplification following a melting curve shows any side products in the reaction. The latter assay is especially important for the SYBR Green method because of its nonspecific binding to any dsDNA product. Also, the uncontrolled poly-A reaction may slightly affect Tm of PCR product. It is therefore important to use a suitable gene, or a geometric mean of multiple reference genes, to normalized qPCR data [143,144]. The most widely used reference gene is snRNA species such as U6. With proper experimental design, qPCR can be a reliable and sensitive method for detection of EBV miRNAs in cells or EVs.

### 5.2. DNA Probes

Molecular beacon (MB) is the first approach to utilize a kind of specially designed DNA probe. MB is a short hairpin oligonucleotide with a fluorophore and a quencher at each end. Under the stem-loop structure, fluorophore is quenched by proximal effects, which could be abolished by hybridizing with its target miRNA [145]. Surface plasmon resonance based method also utilizes DNA probe hybridization and will be discussed in the next section.

As a chemical synthesized oligo, MB could be modified or engineered which greatly expands its applications, even for use in living cells [146]. For miRNAs inside EVs, MBs have been reported to be capable of penetrating EV membranes and hybridizing with its target miR. For example, miR-21 in EVs isolated from breast cancer cell lines were successfully detected by Lee in 2015 [147]. Disruption of EV membranes with streptolysion O resulted in enhanced fluorescent signal likely by delivering more MBs into EVs. In addition, miR-21 inside of cancer cell-derived EVs was selectively detected among heterogeneous EV mixtures and in human serum. Multiple miRNAs can also be detected simultaneously from the same EV preparation using this method [148]. Another group developed a method of encapsulating MBs into positively charged lipoplex nanoparticles [149]. In their study, negatively charged EVs derived from cell membrane were able to fuse with MB containing nanoparticles. This lipoplex-EV fusion leads to the mixing of EV RNAs with MBs and fluorescence signals of MBs were observed by the total internal reflection fluorescence (TIRF) microscopy. This method provides results similar to RT-qPCR for miR-21 detection and is capable of detecting a single biomolecule within the nanocomplexes. Serum can be applied directly on the biochip which is immobilized with MB encapsulated lipoplex nanoparticles. Similar MB tools have also been successfully used in a fluorescent-NTA assay [150].

Another approach relies on the sensitive and specific hybridization of the target miRNA cDNA product to a microarray immobilized with a complementary DNA probe [151,152]. Then the hybridization can be detected by fluorescence or enzyme related detection methods. The principle of the design allows the assay to be performed on hundreds of miRNAs simultaneously from one sample for relatively low cost. However, this approach does have its limitations. First, a probe-based microarray method is semiquantitative and relatively suitable for miRNAs expression level comparison between different conditions. Therefore, quantitative or more reliable assays, like RT-qPCR, are needed for verification purposes. Second, a microarray usually has a smaller dynamic range compared to other profiling methods such as NGS. This compression could underestimate the abundance of some miRNAs and give misleading results [153]. Finally, hybridization-based methods are often unable to distinguishable between some closely related miRNAs [154].

One of the most frequently used methods of miRNA labeling is through an enzymatic attachment reaction (as described in Section 4.1). T4 RNA ligase can be exploited to attach a fluorescently labeled nucleotide or short oligonucleotide to the 3-end of target miRNA. An alternative approach is to use a bridge oligonucleotide with poly-T segment which hybridizes the poly-A tail added by PAP to 3-end of miRNAs. The polymerization reaction by PAP is not well controlled and a variable number of adenosines may be added to the miRNA tail, possibly affecting hybridization as discussed in Section 4.1. Another limitation of this method is the substrate structural preference of T4 RNA ligase could introduce artifact and bias into miRNA detection [155,156]. Lee et al. tried to developed another approach to replace the labeling step in miRNA detection [157]. Biotin-labeled structure-specific RNA binding protein (PAZ-dsRBD derived from Argonaute proteins) is used in this new assay, which can only recognize miRNAs binding with the DNA probe on the array. These probes are designed to require base-stacking stabilization provided by the target miRNA bound.

The RNA-primed array-based Klenow enzyme assay is another method frequently used to remove the miRNA labeling [158]. The probes used for capturing miRNAs consist of an antisense sequence from target miRNA and a universal spacer, three thymidines. After hybridization, single stranded probes are digested by exonuclease I, following the addition of biotinylated adenosine by the Klenow fragment of DNA polymerase I that utilizes the bound miRNA as a primer. The incorporated biotin can then be detected with fluorescent- or enzyme-conjugated streptavidin. A recent publication used a similar approach named ligase-assisted sandwich hybridization, pushing the detection limit to the 30 fM level [159,160]. These data are close to the sensitivity of detection using 1 μg total RNA input in blood samples by RT-qPCR. Currently, most available commercial microarrays are label-based methods and require on the order of tens to hundreds of ng of total RNA input, exhibiting nM to pM detection limits.

### 5.3. NGS

NGS technology is becoming the leading methodology in miRNA research. It has extraordinary profiling ability unlike any other available technology. However, obtaining useful data requires specialized expertise to perform the experiment and to analyze the large data sets produced. NGS also requires expensive reagents and equipment, which can restrict its use. NGS of RNA (also called deep sequencing or RNA-seq) is perhaps the only technique that can exposes the immense variation inherent in miRNA processing and result in the discovery of novel miRNAs or other RNA species. The heterogeneity of miRNA (e.g., iso-miRNA or single-nucleotide changes) can be problematic for other techniques, because the exact miRNA sequence must be known before using RT-qPCR or DNA probe. Moreover, NGS data may include other small RNAs, as piRNA, snoRNA, lnc RNA, yRNA, or even rRNAs [161]. However, because of its ultra-sensitivity in RNA profiling, researchers must be careful when handling EV samples that may introduce contamination and bias at every step.

The two leading NGS platforms are supplied by Illumina and SOLiD [162]. Presently, Illumina is the most widely used sequencing platform for deep sequencing and RNA profiling. This system takes advantage of a fluorescence-based paradigm to read the nucleotides. Ion Torrent from ThermoFisher is another option, which measures pH during polymerization on a semiconductor chip to determine nucleotide sequences.

In addition, there are complications that must be considered when conducting NGS experiments. As with many miRNA profiling tools [163], some of the current miR-seq technology and protocols are not able to accurately determine miRNA expression profiles because the number of reads determined for any given miRNA molecule do not necessarily reflect its actual abundance. This problem may be resolved by using UMI (Unique Molecular Identifier) in which each individual miRNA molecule is tagged with an UMI during library construction [164]. Additionally, the ability and efficiency of current technologies to capture miRNA and other small RNA species has been shown to heavily depend not only on miRNA sequence, but also on library preparation methods [165]. Huang et al. evaluated different commercial small RNA library preparation protocols from three human plasma-derived exosomal RNA samples [166]. Their results show that all the commercial kits generate sufficient DNA fragments and have significant bias of specific small RNAs.

### 5.4. Surface Plasmon Resonance (SPR)

SPR is the oscillation of conduction electrons at the interface of material stimulated by plane-polarized incident light. The electromagnetic surface waves produced results in a direction parallel to the interface, making it very sensitive to any change of this boundary. Based on these principles, SPR is useful for the detection of molecules in solution or surface proteins of whole EVs [167].

Considering the size of miRNA molecules (6–8 nm) and the typical sensing depth of SPR (100–200 nm), unlabeled miRNA can only be detected down to a concentration of 1 nM. Therefore, a signal amplification step is usually required [16,168,169]. Many groups have pushed the detection limit to the sub-fM level by using multiple signal amplification methods.

One of the first applications of SPR-based microarray detection of miRNAs was described for miR-16, miR-23b, and miR-122b [170]. After binding to immobilized probes on the surface, PAP was used to add poly-A tails to the 3-end of the miRNAs. Next, gold nanoparticles were conjugated with poly-T and used to amplify the SPR signal. Using this approach, a 10 fM detection limit was achieved, which is an approximately 10^5^-fold enhancement compared with unlabeled method. In another work, biotin–streptavidin binding was used instead of nanoparticles for signal amplification produced similar results [171]. Another improvement of this approach has been investigated by other groups [172,173]. Biotinylated DNA probes were designed as molecular beacons that only expose its biotin groups after hybridization of target miRNAs. Under optimal conditions, they obtained a limit of detection of 45 fM. Taken together, SPR offers another sensitive method for miRNA detection on a microarray platform.

### 5.5. Localized Surface Plasmon Resonance (LSPR)

When the SPR is confined in a nanoparticle of size comparable to or smaller than the wavelength of light used to excite the plasmon, it generates a localized surface plasmon resonance (LSPR) effect. Under LSPR, electric fields near the nanoparticle’s surface are greatly enhanced and maximize the plasmon resonant frequency. The resonance even falls into visible wavelengths for noble metal nanoparticles [174]. Since the sensing depth of LSPR (5–20 nm) is close to the length of miRNA molecules, direct detection is possible even without any labeling or signal amplification probe. The first report of this method was using the surface of gold nanoprisms in the detection of miR-21 and miR-10b from the plasma of patients with pancreatic cancer [175]. They achieved a sub-fM level detection limit, which is about 2-fold higher than RT-qPCR method. Significantly, miR-21 in plasma samples from Pancreatic ductal adenocarcinoma (PDAC) patients could be detected directly with this method without any purification step which minimize the loss during RNA isolation. The plasmonic biosensor is stable and reusable for several days without compromising sensitivity and selectivity, suggesting it may enable the development of simple, cost-effective tools for diagnostic detection of miRs.

Nanostructures on solid supports are being constructed using current microfabrication approaches. Fabrication of transferring side edge prefunctionalized (SEPF) nanostructure arrays onto flexible substrates (i.e., PET) using contact transfer printing increases sensitivity and reduces background noise [176]. The nanostructure sidewalls have been biochemically synthesized with functional terminals for miRNA hybridization and the immobilization of resonant nanoparticles. This unique configuration has shown a 10 fM level detection capacity of the miR-21 oligonucleotide.

Guo et al. developed a new approach for visual detection of miRNAs in human serum with the naked eye [177]. The target miRNAs were firstly hybridized with the cDNAs in solution, then duplex-specific nuclease (DSN) would specifically cleave the DNA strand and keep miRNA intact. On the next step, the remaining cDNAs are hybridized with two designed probes to form a Y-shaped DNA duplex that assemble the gold nanoparticles on the other side of probes into aggregates. The nanoparticle dimers generated produce a significant plasmon coupling effect correlating with miRNA input amount causing the solution to change color. The corresponding limit of detection is 0.5 fM when inspected with the naked eye, and down to 23 aM with a spectrometer. Therefore, LSPR is likely to be very useful in the detection of miRNA in the clinical samples.

### 5.6. Surface-Enhanced Raman Scattering (SERS)

Surface-enhanced Raman scattering (SERS) is a kind of SPR technique that enhances Raman scattering of molecules adsorbed on solid surfaces or nanostructures. The enhancement factor can be as much as 10^10^ to 10^11^, which constitutes a quantitative fundamental test of single molecules [178]. With application of signal by SERS, miRNAs can be directly detected on a SERS active substrate or indirectly with the aid of nanoprobes. Demonstrated by Driskell et al. [179], near-real time (10 s) detection of miRNAs was archived on OAD-fabricated silver nanorod arrays. As shown in another paper [180], both direct and indirect methods based on SERS can be used to detect miR-21 with the limit of detection of 0.36 and 0.85 nM, respectively.

## 6. Conclusions

Although EVs were first identified in the 1980s, studies on EVs have increased at a remarkable rate in the last five years, especially following the discovery of functional mRNAs and miRNAs in EVs. EVs play a key role in the process of cell-to-cell communication and influence the physiology of recipient cells. With the discovery that exosomal miRNAs are functional in recipient cells, many new questions arise: how are biologically active molecules packaged into EVs for secretion? How are EVs and their components targeted to recipient cells and what are the molecular and phenotypic consequences of EV intercellular transport? The study of viral miRNAs, like those produced by EBV, is likely to help clarify many of the basic mechanisms of EV biology. The most compelling, but challenging application, will be to utilize EVs and their cargo as clinical tools for diagnosis, drug deliver, and gene therapy. The rapid development of new methods of EV isolation and miRNA detection will only continue to push the field forward and lead to new exciting discoveries.

## Figures and Tables

**Table 1:** EBV encoded miRNA expression in associated cancers.

Tumor Type	Sample	Latency Type	BHRF1 Cluster	BART Cluster 1	BART Cluster 2	BART-2	Method	Ref.
BL	MUTUI/ OUS/ BL41/95, Marm. B95.8	I/ II/ III	−/ +/ +	1		+	Northern blot	[35]
	BC-1/ Jijoye/ Raji/ BL41/95/ MUTU III, Namalwa	I/ III/ III/ III/ III	−/ 1-2/ 1-1, 1-2/ 1-1, 1-2/ 1-1, 1-2/	1, 3, 5/ 1, 3, 5/ −/ 1, 3/ 1, 3, 5/	7, 10, 12/ 7, 10, 12/ −/ −/ 7, 10, 12/		Northern blot	[66]
	Jijoye	III		3, 4, 5, 6, 15, 17	7, 8, 9, 10, 11, 12, 13, 14, 19, 20		Northern blot, microarray	[67]
	BL-5, Savl, KemI, Akata, Dante, Daudi/ OkuI, GG68, Raji, MutuIII	I/ III	−/ +	3, 4, 1-5p, 15	7, 10, 12, 20-5p		qPCR	[54]
	2A8.1, RaeI		+				qPCR	[52]
	B95.8-transformed cells/ Daudi/ Namalwa B	I/ I/ III		15/ 15, 16/ 16	−/ 22/ 22		qPCR	[33]
HL	RPMI6666	II	+	1		+	Northern blot	[35]
NPC	C666-1	II	−	3, 4, 1-5p, 15	7, 10, 12, 20-5p		qPCR	[54]
	C666	II	−	1, 3, 5	7, 10, 12		Northern blot	[66]
	C666-1	II	1-1, 1-2, 1-3	+	+	+	qPCR	[52]
	C666-1	II		+	+	+	qPCR	[70]
	C-15		−	1, 3, 5	7, 10, 12		Northern blot	[66]
	clinical tissue; C666; NP460hTERT + EBV			1-3p, 5, 6-5p, 6-3p,17-5p	7, 8, 9, 14,18-5p, 19-3p	+	Microarray, qPCR	[38]
	clinical tissue/ HK1-EBV, C666-1		−	+/ 15, 16	+/ 22	+	qPCR, Deep sequencing	[33]
	clinical tissue		−	+	+	+	qPCR, Deep sequencing	[52]
	clinical tissue		−	+	+		Northern blot, Deep sequencing	[53]
GC	SNU-719	I	−	3, 4, 1-5p, 15	7, 10, 12, 20-5p		qPCR	[54]
	SNU-719	I	−	+	+	+	qPCR	[69]
	AGS-EBV	I		3, 1-3p, 5, 17-5p	7, 9		qPCR	[68]
	clinical tissue, SNU-719			+	+	+	qPCR	[70]
	clinical tissue		−	+	+	+	qPCR	[69]
	YCCEL1	I	−	1-3p, 15-3p	9-3p, 5-5p, 7-3p, 22-3p, 19-3p		Northern blot, qPCR	[71]
	SNU-719, AGS-EBV/ Animal model/ clinical tissue	I/ / /	−/ −/ −/	1, 3, 5/ 1, 5/ 1	7, 10, 12/ 7, 10, 12/ /	+/ / +	Northern blot	[44]
NKTL	Lymphoma clinical tissue		−	+	+	+	Deep sequencing	[65]
LCL	721, B958IID6	III	+	3, 4, 1-5p, 15	−		qPCR	[54]
	IM-9	III	1-1, 1-2	1, 3, 5/	7, 10, 12/		Northern blot	[66]
	IM-9	III		+	+	+	qPCR	[70]
	AT		+				qPCR	[52]
DLBCL	clinical tissue		−	+	+	+	Deep sequencing	[64]
PTLD	PTLD1	III	+	3, 4, 1-5p, 15	7, 10, 12, 20-5p		qPCR	[54]

**Table 2:** Comparison of EV Isolation Methods.

Method	Sample Volume	Yield	Purity	Cost	Advantage	Disadvantage
UC	1–100 mL	++	++	+	No chemical additives	Time and labor intensive; Low throughput; EVs/protein aggregates
UF	Variety	++	+	++	Flexible volume; No chemical additives	Low purity; Low throughput; Efficiency dependent on the type of ultra-membrane
Precipitation	Variety	+++	+	+	Flexible volume; Time and labor saving; No expensive equipment needed	Low purity; Sample contamination by polymer particles; Co-isolation of nonspecific proteins; EV/protein aggregates
SEC	<10 mL	++	+++	+	High purity; Time saving; No chemical additives; Physiological buffer	Sample volume limited; Low throughput; Sample diluted
Immunoaffinity	<5 mL	+	+++	+++	High purity; Physiological buffer; Integration with downstream biological analysis	Sample volume limited; Low throughput; Very selective; Dependent on antibody/protein; Contaminated with antibody/protein; Pre-enrichment needed
AIEX	Variety	++	++	++	Label free; Flexible volume	Low throughput; Sample diluted
Microfluidic	<1 mL	++	N.A.	+++	Label free; Integration with downstream biological analysis	Sample volume extremely limited; Low throughput

## Abbreviations

AIEX	Anion exchange chromatography
BART	BamHI A rightward transcripts
BHRF	BamHI fragment H rightward open reading frame
BL	Burkitt′s lymphoma
DC	Differential ultracentrifugation
DLBCL	Diffuse large B-cell lymphoma
EBV	Epstein–Barr-virus
EV	Extracellular vesicle
GC	Gastric carcinoma
HL	Hodgkin’s lymphoma
LCL	Lymphoblastoid cell line
LSPR	Localized surface plasmon resonance
MB	Molecular beacon
NGS	Next-generation sequencing
NKTL	Nasal NK/T-cell lymphoma
NPC	Nasopharyngeal carcinoma
NTA	Nanoparticle tracking analysis
PAP	Poly(A) polymerase
PTLD	Post-transplant lymphoproliferative disease
RT-qPCR	Reverse transcription quantitative polymerase chain reaction
SEC	Size-exclusion chromatography
SERS	Surface-enhanced Raman scattering
SPR	Surface plasmon resonance
TFF	Tangential flow filtration
UC	Ultracentrifugation
UF	Ultrafiltration
UMI	Unique Molecular Identifier

## References

[1] Lee Y., El Andaloussi S., Wood M.J.A. (2012). Exosomes and microvesicles: Extracellular vesicles for genetic information transfer and gene therapy. Hum. Mol. Gen..

[2] Raposo G., Stoorvogel W. (2013). Extracellular vesicles: Exosomes, microvesicles, and friends. J. Cell Biol..

[3] Akers J.C., Gonda D., Kim R., Carter B.S., Chen C.C. (2013). Biogenesis of extracellular vesicles (EV): Exosomes, microvesicles, retrovirus-like vesicles, and apoptotic bodies. J. Neurooncol..

[4] Szatanek R., Baran J., Siedlar M., Baj-Krzyworzeka M. (2015). Isolation of extracellular vesicles: Determining the correct approach (Review). Int. J. Mol. Med..

[5] Ratajczak J., Wysoczynski M., Hayek F., Janowska-Wieczorek A., Ratajczak M.Z. (2006). Membrane-derived microvesicles: Important and underappreciated mediators of cell-to-cell communication. Leukemia.

[6] Robbins P.D., Morelli A.E. (2014). Regulation of immune responses by extracellular vesicles. Nat. Rev. Immunol..

[7] Camussi G., Deregibus M.C., Bruno S., Cantaluppi V., Biancone L. (2010). Exosomes/microvesicles as a mechanism of cell-to-cell communication. Kidney Int..

[8] Nishida-Aoki N., Ochiya T. (2015). Interactions between cancer cells and normal cells via miRNAs in extracellular vesicles. Cell. Mol. Life Sci..

[9] Zhang Y., Yang P., Wang X.F. (2014). Microenvironmental regulation of cancer metastasis by miRNAs. Trends Cell Biol..

[10] Mei Q., Li X., Guo M., Fu X., Han W. (2014). The miRNA network: Micro-regulator of cell signaling in cancer. Expert Rev. Anticancer Ther..

[11] Si M., Zhu S., Wu H., Lu Z., Wu F., Mo Y. (2007). miR-21-mediated tumor growth. Oncogene.

[12] Baranwal S., Alahari S.K. (2010). miRNA control of tumor cell invasion and metastasis. Int. J. Cancer.

[13] Witwer K.W. (2015). Circulating microRNA biomarker studies: Pitfalls and potential solutions. Clin. Chem..

[14] Shin V.Y., Chu K.M. (2014). MiRNA as potential biomarkers and therapeutic targets for gastric cancer. World J. Gastroenterol..

[15] Javidi M.A., Ahmadi A.H., Bakhshinejad B., Nouraee N., Babashah S., Sadeghizadeh M. (2014). Cell-free microRNAs as cancer biomarkers: The odyssey of miRNAs through body fluids. Med. Oncol..

[16] Hayes J., Peruzzi P.P., Lawler S. (2014). MicroRNAs in cancer: Biomarkers, functions and therapy. Trends Mol. Med..

[17] Anastasiadou E., Jacob L.S., Slack F.J. (2018). Non-coding RNA networks in cancer. Nat. Rev. Cancer.

[18] Adams B.D., Anastasiadou E., Esteller M., He L., Slack F.J. (2015). The inescapable influence of noncoding RNAs in cancer. Cancer Res..

[19] Pfeffer S.R., Yang C.H., Pfeffer L.M. (2015). The Role of miR-21 in Cancer. Drug Dev. Res..

[20] Stott S.L., Hsu C.-H., Tsukrov D.I., Yu M., Miyamoto D.T., Waltman B.A., Rothenberg S.M., Shah A.M., Smas M.E., Korir G.K. (2010). Isolation of circulating tumor cells using a microvortex-generating herringbone-chip. Proc. Natl. Acad. Sci. USA.

[21] Davis J.W., Nakanishi H., Kumar V.S., Bhadkamkar V.A., McCormack R., Fritsche H.A., Handy B., Gornet T., Babaian R.J. (2008). Circulating tumor cells in peripheral blood samples from patients with increased serum prostate specific antigen: Initial results in early prostate cancer. J. Urol..

[22] Lobb R.J., Becker M., Wen S.W., Wong C.S., Wiegmans A.P., Leimgruber A., Moller A. (2015). Optimized exosome isolation protocol for cell culture supernatant and human plasma. J. Extracell. Vesicles.

[23] Malloci M., Perdomo L., Veerasamy M., Andriantsitohaina R., Simard G., Martinez M.C. (2018). Extracellular Vesicles: Mechanisms in Human Health and Disease. Antioxid Redox Signal..

[24] Maia J., Caja S., Strano Moraes M.C., Couto N., Costa-Silva B. (2018). Exosome-Based Cell-Cell Communication in the Tumor Microenvironment. Front. Cell Dev. Biol.

[25] Katsuda T., Kosaka N., Ochiya T. (2014). The roles of extracellular vesicles in cancer biology: Toward the development of novel cancer biomarkers. Proteomics.

[26] Sadovska L., Eglitis J., Line A. (2015). Extracellular Vesicles as Biomarkers and Therapeutic Targets in Breast Cancer. Anticancer Res..

[27] Boukouris S., Mathivanan S. (2015). Exosomes in bodily fluids are a highly stable resource of disease biomarkers. Proteom. Clin. Appl..

[28] Tian F., Shen Y., Chen Z., Li R., Ge Q. (2017). No Significant Difference between Plasma miRNAs and Plasma-Derived Exosomal miRNAs from Healthy People. Biomed. Res. Int..

[29] Ge Q., Zhou Y., Lu J., Bai Y., Xie X., Lu Z.J.M. (2014). miRNA in plasma exosome is stable under different storage conditions. Molecules.

[30] Epstein M.A., Achong B.G., Barr Y.M. (1964). Virus Particles in Cultured Lymphoblasts from Burkitt’s Lymphoma. Lancet.

[31] Thorley-Lawson D.A. (2005). EBV the prototypical human tumor virus–just how bad is it?. J. Allergy Clin. Immunol..

[32] Masucci M.G., Ernberg I. (1994). Epstein-Barr virus: Adaptation to a life within the immune system. Trends Microbiol..

[33] Chen S.J., Chen G.H., Chen Y.H., Liu C.Y., Chang K.P., Chang Y.S., Chen H.C. (2010). Characterization of Epstein-Barr virus miRNAome in nasopharyngeal carcinoma by deep sequencing. PLoS ONE.

[34] Edwards R.H., Marquitz A.R., Raab-Traub N.J.J.O.V. (2008). Epstein-Barr virus BART microRNAs are produced from a large intron prior to splicing. J. Virol..

[35] Pfeffer S., Zavolan M., Grasser F.A., Chien M., Russo J.J., Ju J., John B., Enright A.J., Marks D., Sander C. (2004). Identification of virus-encoded microRNAs. Science.

[36] Choy E.Y., Siu K.L., Kok K.H., Lung R.W., Tsang C.M., To K.F., Kwong D.L., Tsao S.W., Jin D.Y. (2008). An Epstein-Barr virus-encoded microRNA targets PUMA to promote host cell survival. J. Exp. Med..

[37] Barth S., Meister G., Grasser F.A. (2011). EBV-encoded miRNAs. Biochim. Biophys. Acta.

[38] Wong A.M., Kong K.L., Tsang J.W., Kwong D.L., Guan X.Y. (2012). Profiling of Epstein-Barr virus-encoded microRNAs in nasopharyngeal carcinoma reveals potential biomarkers and oncomirs. Cancer.

[39] Cullen B.R. (2009). Viral and cellular messenger RNA targets of viral microRNAs. Nature.

[40] Lo A.K., To K.F., Lo K.W., Lung R.W., Hui J.W., Liao G., Hayward S.D. (2007). Modulation of LMP1 protein expression by EBV-encoded microRNAs. Proc. Natl. Acad. Sci. USA.

[41] Barth S., Pfuhl T., Mamiani A., Ehses C., Roemer K., Kremmer E., Jaker C., Hock J., Meister G., Grasser F.A. (2008). Epstein-Barr virus-encoded microRNA miR-BART2 down-regulates the viral DNA polymerase BALF5. Nucleic Acids Res..

[42] Lung R.W., Tong J.H., Sung Y.M., Leung P.S., Ng D.C., Chau S.L., Chan A.W., Ng E.K., Lo K.W., To K.F. (2009). Modulation of LMP2A expression by a newly identified Epstein-Barr virus-encoded microRNA miR-BART22. Neoplasia.

[43] Nachmani D., Stern-Ginossar N., Sarid R., Mandelboim O. (2009). Diverse herpesvirus microRNAs target the stress-induced immune ligand MICB to escape recognition by natural killer cells. Cell Host Microbe.

[44] Kim D.N., Chae H.S., Oh S.T., Kang J.H., Park C.H., Park W.S., Takada K., Lee J.M., Lee W.K., Lee S.K. (2007). Expression of viral microRNAs in Epstein-Barr virus-associated gastric carcinoma. J. Virol..

[45] Iizasa H., Wulff B.E., Alla N.R., Maragkakis M., Megraw M., Hatzigeorgiou A., Iwakiri D., Takada K., Wiedmer A., Showe L. (2010). Editing of Epstein-Barr virus-encoded BART6 microRNAs controls their dicer targeting and consequently affects viral latency. J. Biol. Chem..

[46] Anastasiadou E., Garg N., Bigi R., Yadav S., Campese A.F., Lapenta C., Spada M., Cuomo L., Botta A., Belardelli F. (2015). Epstein-Barr virus infection induces miR-21 in terminally differentiated malignant B cells. Int J. Cancer.

[47] Tagawa T., Albanese M., Bouvet M., Moosmann A., Mautner J., Heissmeyer V., Zielinski C., Lutter D., Hoser J., Hastreiter M. (2016). Epstein-Barr viral miRNAs inhibit antiviral CD4+ T cell responses targeting IL-12 and peptide processing. J. Exp. Med..

[48] Albanese M., Tagawa T., Bouvet M., Maliqi L., Lutter D., Hoser J., Hastreiter M., Hayes M., Sugden B., Martin L. (2016). Epstein-Barr virus microRNAs reduce immune surveillance by virus-specific CD8+ T cells. Proc. Natl. Acad. Sci. USA.

[49] Seto E., Moosmann A., Gromminger S., Walz N., Grundhoff A., Hammerschmidt W. (2010). Micro RNAs of Epstein-Barr virus promote cell cycle progression and prevent apoptosis of primary human B cells. PLoS Pathog..

[50] Albanese M., Tagawa T., Buschle A., Hammerschmidt W. (2017). MicroRNAs of Epstein-Barr Virus Control Innate and Adaptive Antiviral Immunity. J. Virol..

[51] Kim H., Choi H., Lee S.K. (2015). Epstein-Barr virus miR-BART20-5p regulates cell proliferation and apoptosis by targeting BAD. Cancer Lett..

[52] Cosmopoulos K., Pegtel M., Hawkins J., Moffett H., Novina C., Middeldorp J., Thorley-Lawson D.A. (2009). Comprehensive profiling of Epstein-Barr virus microRNAs in nasopharyngeal carcinoma. J. Virol..

[53] Zhu J.Y., Pfuhl T., Motsch N., Barth S., Nicholls J., Grasser F., Meister G. (2009). Identification of novel Epstein-Barr virus microRNA genes from nasopharyngeal carcinomas. J. Virol..

[54] Pratt Z.L., Kuzembayeva M., Sengupta S., Sugden B. (2009). The microRNAs of Epstein-Barr Virus are expressed at dramatically differing levels among cell lines. Virology.

[55] Wei W.I., Sham J.S.T. (2005). Nasopharyngeal carcinoma. Lancet.

[56] Sheng L., Shui Y., Shen L., Wei Q. (2008). Effect of patient-related delay in diagnosis on the extent of disease and prognosis in nasopharyngeal carcinoma. Am. J. Rhinol..

[57] Arango B.A., Castrellon A.B., Perez C.A., Raez L.E., Santos E.S. (2010). Nasopharyngeal carcinoma: Alternative treatment options after disease progression. Expert Rev. Anticancer Ther..

[58] Kefas B., Comeau L., Floyd D.H., Seleverstov O., Godlewski J., Schmittgen T., Jiang J., Li Y., Chiocca E.A., Lee J. (2009). The neuronal microRNA miR-326 acts in a feedback loop with notch and has therapeutic potential against brain tumors. J. Neurosci..

[59] Jiang X., Hu C., Arnovitz S., Bugno J., Yu M., Zuo Z., Chen P., Huang H., Ulrich B., Gurbuxani S. (2016). miR-22 has a potent anti-tumour role with therapeutic potential in acute myeloid leukaemia. Nat. Commun..

[60] Fesler A., Liu H., Ju J. (2018). Modified miR-15a has therapeutic potential for improving treatment of advanced stage colorectal cancer through inhibition of BCL2, BMI1, YAP1 and DCLK1. Oncotarget.

[61] Zhang G., Zong J., Lin S., Verhoeven R.J., Tong S., Chen Y., Ji M., Cheng W., Tsao S.W., Lung M. (2015). Circulating Epstein-Barr virus microRNAs miR-BART7 and miR-BART13 as biomarkers for nasopharyngeal carcinoma diagnosis and treatment. Int. J. Cancer.

[62] He M.L., Luo M.X., Lin M.C., Kung H.F. (2012). MicroRNAs: Potential diagnostic markers and therapeutic targets for EBV-associated nasopharyngeal carcinoma. Biochim. Biophys. Acta.

[63] Liu X., Luo H.N., Tian W.D., Lu J., Li G., Wang L., Zhang B., Liang B.J., Peng X.H., Lin S.X. (2013). Diagnostic and prognostic value of plasma microRNA deregulation in nasopharyngeal carcinoma. Cancer Biol. Ther..

[64] Imig J., Motsch N., Zhu J.Y., Barth S., Okoniewski M., Reineke T., Tinguely M., Faggioni A., Trivedi P., Meister G. (2011). microRNA profiling in Epstein-Barr virus-associated B-cell lymphoma. Nucleic Acids Res..

[65] Motsch N., Alles J., Imig J., Zhu J., Barth S., Reineke T., Tinguely M., Cogliatti S., Dueck A., Meister G. (2012). MicroRNA profiling of Epstein-Barr virus-associated NK/T-cell lymphomas by deep sequencing. PLoS ONE.

[66] Cai X., Schafer A., Lu S., Bilello J.P., Desrosiers R.C., Edwards R., Raab-Traub N., Cullen B.R. (2006). Epstein-Barr virus microRNAs are evolutionarily conserved and differentially expressed. PLoS Pathog..

[67] Grundhoff A., Sullivan C.S., Ganem D. (2006). A combined computational and microarray-based approach identifies novel microRNAs encoded by human gamma-herpesviruses. RNA.

[68] Marquitz A.R., Mathur A., Shair K.H., Raab-Traub N. (2012). Infection of Epstein-Barr virus in a gastric carcinoma cell line induces anchorage independence and global changes in gene expression. Proc. Natl. Acad. Sci. USA.

[69] Shinozaki-Ushiku A., Kunita A., Isogai M., Hibiya T., Ushiku T., Takada K., Fukayama M. (2015). Profiling of Virus-Encoded MicroRNAs in Epstein-Barr Virus-Associated Gastric Carcinoma and Their Roles in Gastric Carcinogenesis. J. Virol..

[70] Tsai C.Y., Liu Y.Y., Liu K.H., Hsu J.T., Chen T.C., Chiu C.T., Yeh T.S. (2017). Comprehensive profiling of virus microRNAs of Epstein-Barr virus-associated gastric carcinoma: Highlighting the interactions of ebv-Bart9 and host tumor cells. J. Gastroenterol. Hepatol..

[71] Kim D.N., Seo M.K., Choi H., Kim S.Y., Shin H.J., Yoon A.R., Tao Q., Rha S.Y., Lee S.K. (2013). Characterization of naturally Epstein-Barr virus-infected gastric carcinoma cell line YCCEL1. J. Gen. Virol..

[72] Qiu J., Cosmopoulos K., Pegtel M., Hopmans E., Murray P., Middeldorp J., Shapiro M., Thorley-Lawson D.A. (2011). A novel persistence associated EBV miRNA expression profile is disrupted in neoplasia. PLoS Pathog..

[73] Amoroso R., Fitzsimmons L., Thomas W.A., Kelly G.L., Rowe M., Bell A.I. (2011). Quantitative studies of Epstein-Barr virus-encoded microRNAs provide novel insights into their regulation. J. Virol..

[74] Meckes D.G. (2015). Exosomal communication goes viral. J. Virol..

[75] Meckes D.G., Gunawardena H.P., Dekroon R.M., Heaton P.R., Edwards R.H., Ozgur S., Griffith J.D., Damania B., Raab-Traub N. (2013). Modulation of B-cell exosome proteins by gamma herpesvirus infection. Proc. Natl. Acad. Sci. USA.

[76] Pegtel D.M., Cosmopoulos K., Thorley-Lawson D.A., van Eijndhoven M.A., Hopmans E.S., Lindenberg J.L., de Gruijl T.D., Wurdinger T., Middeldorp J.M. (2010). Functional delivery of viral miRNAs via exosomes. Proc. Natl. Acad. Sci. USA.

[77] Canitano A., Venturi G., Borghi M., Ammendolia M.G., Fais S. (2013). Exosomes released in vitro from Epstein-Barr virus (EBV)-infected cells contain EBV-encoded latent phase mRNAs. Cancer Lett..

[78] Gourzones C., Gelin A., Bombik I., Klibi J., Vérillaud B., Guigay J., Lang P., Témam S., Schneider V., Amiel C. (2010). Extra-cellular release and blood diffusion of BART viral micro-RNAs produced by EBV-infected nasopharyngeal carcinoma cells. Virol. J..

[79] Meckes D.G., Shair K.H., Marquitz A.R., Kung C.P., Edwards R.H., Raab-Traub N. (2010). Human tumor virus utilizes exosomes for intercellular communication. Proc. Natl. Acad. Sci. USA.

[80] Aga M., Bentz G.L., Raffa S., Torrisi M.R., Kondo S., Wakisaka N., Yoshizaki T., Pagano J.S., Shackelford J. (2014). Exosomal HIF1alpha supports invasive potential of nasopharyngeal carcinoma-associated LMP1-positive exosomes. Oncogene.

[81] Nanbo A., Kawanishi E., Yoshida R., Yoshiyama H. (2013). Exosomes derived from Epstein-Barr virus-infected cells are internalized via caveola-dependent endocytosis and promote phenotypic modulation in target cells. J. Virol..

[82] Gutzeit C., Nagy N., Gentile M., Lyberg K., Gumz J., Vallhov H., Puga I., Klein E., Gabrielsson S., Cerutti A. (2014). Exosomes derived from Burkitt’s lymphoma cell lines induce proliferation, differentiation, and class-switch recombination in B cells. J. Immunol..

[83] Baglio S.R., van Eijndhoven M.A., Koppers-Lalic D., Berenguer J., Lougheed S.M., Gibbs S., Leveille N., Rinkel R.N., Hopmans E.S., Swaminathan S. (2016). Sensing of latent EBV infection through exosomal transfer of 5′pppRNA. Proc. Natl. Acad. Sci. USA.

[84] Rechavi O., Erlich Y., Amram H., Flomenblit L., Karginov F.V., Goldstein I., Hannon G.J., Kloog Y. (2009). Cell contact-dependent acquisition of cellular and viral nonautonomously encoded small RNAs. Genes Dev..

[85] Haneklaus M., Gerlic M., Kurowska-Stolarska M., Rainey A.A., Pich D., McInnes I.B., Hammerschmidt W., O’Neill L.A., Masters S.L. (2012). Cutting edge: MiR-223 and EBV miR-BART15 regulate the NLRP3 inflammasome and IL-1beta production. J. Immunol..

[86] Sadeghipour S., Mathias R.A. (2017). Herpesviruses hijack host exosomes for viral pathogenesis. Semin. Cell Dev. Biol..

[87] Kirschner M.B., Edelman J.J., Kao S.C., Vallely M.P., van Zandwijk N., Reid G. (2013). The Impact of Hemolysis on Cell-Free microRNA Biomarkers. Front. Genet..

[88] Witwer K.W., Buzas E.I., Bemis L.T., Bora A., Lasser C., Lotvall J., Nolte-’t Hoen E.N., Piper M.G., Sivaraman S., Skog J. (2013). Standardization of sample collection, isolation and analysis methods in extracellular vesicle research. J. Extracell. Vesicles.

[89] Thery C., Amigorena S., Raposo G., Clayton A. (2006). Isolation and characterization of exosomes from cell culture supernatants and biological fluids. Curr. Protoc. Cell. Biol..

[90] Redgrave T.G., Roberts D.C., West C.E. (1975). Separation of plasma lipoproteins by density-gradient ultracentrifugation. Anal. Biochem..

[91] Cheruvanky A., Zhou H., Pisitkun T., Kopp J.B., Knepper M.A., Yuen P.S., Star R.A. (2007). Rapid isolation of urinary exosomal biomarkers using a nanomembrane ultrafiltration concentrator. Am. J. Physiol. Renal. Physiol..

[92] Nordin J.Z., Lee Y., Vader P., Mäger I., Johansson H.J., Heusermann W., Wiklander O.P.B., Hällbrink M., Seow Y., Bultema J.J. (2015). Ultrafiltration with size-exclusion liquid chromatography for high yield isolation of extracellular vesicles preserving intact biophysical and functional properties. Nanomedicine.

[93] Corso G., Mäger I., Lee Y., Görgens A., Bultema J., Giebel B., Wood M.J.A., Nordin J.Z., Andaloussi S.E.L. (2017). Reproducible and scalable purification of extracellular vesicles using combined bind-elute and size exclusion chromatography. Sci. Rep..

[94] Heinemann M.L., Ilmer M., Silva L.P., Hawke D.H., Recio A., Vorontsova M.A., Alt E., Vykoukal J. (2014). Benchtop isolation and characterization of functional exosomes by sequential filtration. J. Chromatogr. A.

[95] Pertoft H. (2000). Fractionation of cells and subcellular particles with Percoll. J. Biochem. Biophys. Methods.

[96] Boing A.N., van der Pol E., Grootemaat A.E., Coumans F.A., Sturk A., Nieuwland R. (2014). Single-step isolation of extracellular vesicles by size-exclusion chromatography. J. Extracell. Vesicles.

[97] Welton J.L., Webber J.P., Botos L.A., Jones M., Clayton A. (2015). Ready-made chromatography columns for extracellular vesicle isolation from plasma. J. Extracell. Vesicles.

[98] Koh Y.Q., Almughlliq F.B., Vaswani K., Peiris H.N., Mitchell M.D. (2018). Exosome enrichment by ultracentrifugation and size exclusion chromatography. Front Biosci..

[99] Heath N., Grant L., De Oliveira T.M., Rowlinson R., Osteikoetxea X., Dekker N., Overman R. (2018). Rapid isolation and enrichment of extracellular vesicle preparations using anion exchange chromatography. Sci. Rep..

[100] Pastorek J., Marcinka K. (1989). Effects of pH and ionic strength on precipitation of phytopathogenic viruses by polyethylene glycol. Acta Virol..

[101] Green M.R., Sambrook J. (2017). Preparation of Single-Stranded Bacteriophage M13 DNA by Precipitation with Polyethylene Glycol. Cold Spring Harb. Protoc..

[102] Yamamoto K.R., Alberts B.M., Benzinger R., Lawhorne L., Treiber G. (1970). Rapid bacteriophage sedimentation in the presence of polyethylene glycol and its application to large-scale virus purification. Virology.

[103] Wang K., Zhang S., Weber J., Baxter D., Galas D.J. (2010). Export of microRNAs and microRNA-protective protein by mammalian cells. Nucleic Acids Res..

[104] Hurwitz S.N., Meckes D.G. (2017). An Adaptable Polyethylene Glycol-Based Workflow for Proteomic Analysis of Extracellar Vesicles. Methods Mol. Biol..

[105] Tang Y.T., Huang Y.Y., Zheng L., Qin S.H., Xu X.P., An T.X., Xu Y., Wu Y.S., Hu X.M., Ping B.H. (2017). Comparison of isolation methods of exosomes and exosomal RNA from cell culture medium and serum. Int. J. Mol. Med..

[106] Van Deun J., Mestdagh P., Sormunen R., Cocquyt V., Vermaelen K., Vandesompele J., Bracke M., De Wever O., Hendrix A. (2014). The impact of disparate isolation methods for extracellular vesicles on downstream RNA profiling. J. Extracell. Vesicles.

[107] Rider M.A., Hurwitz S.N., Meckes D.G. (2016). ExtraPEG: A Polyethylene Glycol-Based Method for Enrichment of Extracellular Vesicles. Sci. Rep..

[108] Chen C., Skog J., Hsu C.H., Lessard R.T., Balaj L., Wurdinger T., Carter B.S., Breakefield X.O., Toner M., Irimia D. (2010). Microfluidic isolation and transcriptome analysis of serum microvesicles. Lab. Chip.

[109] Wubbolts R., Leckie R.S., Veenhuizen P.T., Schwarzmann G., Mobius W., Hoernschemeyer J., Slot J.W., Geuze H.J., Stoorvogel W. (2003). Proteomic and biochemical analyses of human B cell-derived exosomes. Potential implications for their function and multivesicular body formation. J. Biol. Chem..

[110] Caby M.P., Lankar D., Vincendeau-Scherrer C., Raposo G., Bonnerot C. (2005). Exosomal-like vesicles are present in human blood plasma. Int. Immunol..

[111] Willms E., Johansson H.J., Mäger I., Lee Y., Blomberg K.E.M., Sadik M., Alaarg A., Smith C.I.E., Lehtiö J., El Andaloussi S. (2016). Cells release subpopulations of exosomes with distinct molecular and biological properties. Sci. Rep..

[112] Kowal J., Arras G., Colombo M., Jouve M., Morath J.P., Primdal-Bengtson B., Dingli F., Loew D., Tkach M., Thery C. (2016). Proteomic comparison defines novel markers to characterize heterogeneous populations of extracellular vesicle subtypes. Proc. Natl. Acad. Sci. USA.

[113] Hurwitz S.N., Rider M.A., Bundy J.L., Liu X., Singh R.K., Meckes D.G. (2016). Proteomic profiling of NCI-60 extracellular vesicles uncovers common protein cargo and cancer type-specific biomarkers. Oncotarget.

[114] Zarovni N., Corrado A., Guazzi P., Zocco D., Lari E., Radano G., Muhhina J., Fondelli C., Gavrilova J., Chiesi A. (2015). Integrated isolation and quantitative analysis of exosome shuttled proteins and nucleic acids using immunocapture approaches. Methods.

[115] Enderle D., Spiel A., Coticchia C.M., Berghoff E., Mueller R., Schlumpberger M., Sprenger-Haussels M., Shaffer J.M., Lader E., Skog J. (2015). Characterization of RNA from Exosomes and Other Extracellular Vesicles Isolated by a Novel Spin Column-Based Method. PLoS ONE.

[116] Nakai W., Yoshida T., Diez D., Miyatake Y., Nishibu T., Imawaka N., Naruse K., Sadamura Y., Hanayama R. (2016). A novel affinity-based method for the isolation of highly purified extracellular vesicles. Sci. Rep..

[117] Balaj L., Atai N.A., Chen W., Mu D., Tannous B.A., Breakefield X.O., Skog J., Maguire C.A. (2015). Heparin affinity purification of extracellular vesicles. Sci. Rep..

[118] Bjork I., Lindahl U. (1982). Mechanism of the anticoagulant action of heparin. Mol. Cell Biochem..

[119] Edward Conrad H. (1998). Heparin-Binding Proteins in Lipoprotein Metabolism. Heparin-Binding Proteins.

[120] Edward Conrad H. (1998). Fibroblast Growth Factors. Heparin-Binding Proteins.

[121] Edward Conrad H. (1998). Heparin-Binding Proteins in Hemostasis. Heparin-Binding Proteins.

[122] Edward Conrad H. (1998). Antithrombin, the Prototypic Heparin-Binding Protein. Heparin-Binding Proteins.

[123] Lee K., Shao H., Weissleder R., Lee H. (2015). Acoustic purification of extracellular microvesicles. ACS Nano.

[124] Liu C., Guo J., Tian F., Yang N., Yan F., Ding Y., Wei J., Hu G., Nie G., Sun J. (2017). Field-Free Isolation of Exosomes from Extracellular Vesicles by Microfluidic Viscoelastic Flows. ACS Nano.

[125] Rekker K., Saare M., Roost A.M., Kubo A.L., Zarovni N., Chiesi A., Salumets A., Peters M. (2014). Comparison of serum exosome isolation methods for microRNA profiling. Clin. Biochem..

[126] Eldh M., Lotvall J., Malmhall C., Ekstrom K. (2012). Importance of RNA isolation methods for analysis of exosomal RNA: Evaluation of different methods. Mol. Immunol..

[127] Buschmann D., Kirchner B., Hermann S., Märte M., Wurmser C., Brandes F., Kotschote S., Bonin M., Steinlein O.K., Pfaffl M.W. (2018). Evaluation of serum extracellular vesicle isolation methods for profiling miRNAs by next-generation sequencing. J. Extracell. Vesicles.

[128] Schageman J., Zeringer E., Li M., Barta T., Lea K., Gu J., Magdaleno S., Setterquist R., Vlassov A.V. (2013). The complete exosome workflow solution: From isolation to characterization of RNA cargo. Biomed. Res. Int..

[129] Stranska R., Gysbrechts L., Wouters J., Vermeersch P., Bloch K., Dierickx D., Andrei G., Snoeck R. (2018). Comparison of membrane affinity-based method with size-exclusion chromatography for isolation of exosome-like vesicles from human plasma. J. Transl. Med..

[130] Andreu Z., Rivas E., Sanguino-Pascual A., Lamana A., Marazuela M., Gonzalez-Alvaro I., Sanchez-Madrid F., de la Fuente H., Yanez-Mo M. (2016). Comparative analysis of EV isolation procedures for miRNAs detection in serum samples. J. Extracell. Vesicles.

[131] Wachalska M., Koppers-Lalic D., van Eijndhoven M., Pegtel M., Geldof A.A., Lipinska A.D., van Moorselaar R.J., Bijnsdorp I.V. (2016). Protein Complexes in Urine Interfere with Extracellular Vesicle Biomarker Studies. J. Circ. Biomark..

[132] Royo F., Diwan I., Tackett M.R., Zuniga P., Sanchez-Mosquera P., Loizaga-Iriarte A., Ugalde-Olano A., Lacasa I., Perez A., Unda M. (2016). Comparative miRNA Analysis of Urine Extracellular Vesicles Isolated through Five Different Methods. Cancers.

[133] Channavajjhala Sarath K., Rossato M., Morandini F., Castagna A., Pizzolo F., Bazzoni F., Olivieri O. (2014). Optimizing the purification and analysis of miRNAs from urinary exosomes. Clin. Chem. Lab. Med..

[134] Hunt E.A., Broyles D., Head T., Deo S.K. (2015). MicroRNA Detection: Current Technology and Research Strategies. Annu. Rev. Anal. Chem..

[135] Lee R.C., Feinbaum R.L., Ambros V. (1993). The *C. elegans* heterochronic gene lin-4 encodes small RNAs with antisense complementarity to lin-14. Cell.

[136] Schmittgen T.D., Lee E.J., Jiang J., Sarkar A., Yang L., Elton T.S., Chen C. (2008). Real-time PCR quantification of precursor and mature microRNA. Methods.

[137] Kramer M.F. (2011). Stem-loop RT-qPCR for miRNAs. Curr. Protoc. Mol. Biol..

[138] Shi R., Chiang V.L. (2005). Facile means for quantifying microRNA expression by real-time PCR. Biotechniques.

[139] Benes V., Collier P., Kordes C., Stolte J., Rausch T., Muckentaler M.U., Häussinger D., Castoldi M. (2015). Identification of cytokine-induced modulation of microRNA expression and secretion as measured by a novel microRNA specific qPCR assay. Sci. Rep..

[140] Li J., Yao B., Huang H., Wang Z., Sun C., Fan Y., Chang Q., Li S., Wang X., Xi J. (2009). Real-Time Polymerase Chain Reaction MicroRNA Detection Based on Enzymatic Stem-Loop Probes Ligation. Anal. Chem..

[141] Zhang J., Li Z., Wang H., Wang Y., Jia H., Yan J. (2011). Ultrasensitive quantification of mature microRNAs by real-time PCR based on ligation of a ribonucleotide-modified DNA probe. Chem. Commun..

[142] Androvic P., Valihrach L., Elling J., Sjoback R., Kubista M. (2017). Two-tailed RT-qPCR: A novel method for highly accurate miRNA quantification. Nucleic Acids Res..

[143] Becker C., Hammerle-Fickinger A., Riedmaier I., Pfaffl M.W. (2010). mRNA and microRNA quality control for RT-qPCR analysis. Methods.

[144] Mestdagh P., Van Vlierberghe P., De Weer A., Muth D., Westermann F., Speleman F., Vandesompele J. (2009). A novel and universal method for microRNA RT-qPCR data normalization. Genome Biol..

[145] Baker M.B., Bao G., Searles C.D. (2011). In vitro quantification of specific microRNA using molecular beacons. Nucleic Acids Res..

[146] Bao G., Rhee W.J., Tsourkas A. (2009). Fluorescent probes for live-cell RNA detection. Annu. Rev. Biomed. Eng..

[147] Lee J.H., Kim J.A., Kwon M.H., Kang J.Y., Rhee W.J. (2015). In situ single step detection of exosome microRNA using molecular beacon. Biomaterials.

[148] Lee J.H., Kim J.A., Jeong S., Rhee W.J. (2016). Simultaneous and multiplexed detection of exosome microRNAs using molecular beacons. Biosens. Bioelectron..

[149] Wu Y., Kwak K.J., Agarwal K., Marras A., Wang C., Mao Y., Huang X., Ma J., Yu B., Lee R. (2013). Detection of extracellular RNAs in cancer and viral infection via tethered cationic lipoplex nanoparticles containing molecular beacons. Anal. Chem..

[150] Baldwin S., Deighan C., Bandeira E., Kwak K.J., Rahman M., Nana-Sinkam P., Lee L.J., Paulaitis M.E. (2017). Analyzing the miRNA content of extracellular vesicles by fluorescence nanoparticle tracking. Nanomedicine.

[151] Liu C.G., Calin G.A., Volinia S., Croce C.M. (2008). MicroRNA expression profiling using microarrays. Nat. Protoc..

[152] Chen Y., Gelfond J.A., McManus L.M., Shireman P.K. (2009). Reproducibility of quantitative RT-PCR array in miRNA expression profiling and comparison with microarray analysis. BMC Genom..

[153] Hesse J., Jacak J., Kasper M., Regl G., Eichberger T., Winklmayr M., Aberger F., Sonnleitner M., Schlapak R., Howorka S. (2006). RNA expression profiling at the single molecule level. Genome Res..

[154] Kane M.D., Jatkoe T.A., Stumpf C.R., Lu J., Thomas J.D., Madore S.J. (2000). Assessment of the sensitivity and specificity of oligonucleotide (50mer) microarrays. Nucleic Acids Res..

[155] Zhuang F., Fuchs R.T., Sun Z., Zheng Y., Robb G.B. (2012). Structural bias in T4 RNA ligase-mediated 3′-adapter ligation. Nucleic Acids Res..

[156] Shen Y., Zheng K.-X., Duan D., Jiang L., Li J. (2012). Label-Free MicroRNA Profiling Not Biased by 3′ End 2′-*O*-Methylation. Anal. Chem..

[157] Lee J.M., Cho H., Jung Y. (2010). Fabrication of a structure-specific RNA binder for array detection of label-free microRNA. Angew. Chem. Int. Ed. Engl..

[158] Nelson P.T., Baldwin D.A., Scearce L.M., Oberholtzer J.C., Tobias J.W., Mourelatos Z. (2004). Microarray-based, high-throughput gene expression profiling of microRNAs. Nat. Methods.

[159] Iizuka R., Ueno T., Funatsu T. (2016). Detection and Quantification of MicroRNAs by Ligase-Assisted Sandwich Hybridization on a Microarray. Methods Mol. Biol..

[160] Ueno T., Funatsu T. (2014). Label-free quantification of microRNAs using ligase-assisted sandwich hybridization on a DNA microarray. PLoS ONE.

[161] van Balkom B.W., Eisele A.S., Pegtel D.M., Bervoets S., Verhaar M.C. (2015). Quantitative and qualitative analysis of small RNAs in human endothelial cells and exosomes provides insights into localized RNA processing, degradation and sorting. J. Extracell. Vesicles.

[162] Lahens N.F., Ricciotti E., Smirnova O., Toorens E., Kim E.J., Baruzzo G., Hayer K.E., Ganguly T., Schug J., Grant G.R. (2017). A comparison of Illumina and Ion Torrent sequencing platforms in the context of differential gene expression. BMC Genom..

[163] Leshkowitz D., Horn-Saban S., Parmet Y., Feldmesser E. (2013). Differences in microRNA detection levels are technology and sequence dependent. RNA.

[164] Islam S., Zeisel A., Joost S., La Manno G., Zajac P., Kasper M., Lönnerberg P., Linnarsson S. (2013). Quantitative single-cell RNA-seq with unique molecular identifiers. Nat. Methods.

[165] van Dijk E.L., Jaszczyszyn Y., Thermes C. (2014). Library preparation methods for next-generation sequencing: Tone down the bias. Exp. Cell Res..

[166] Huang X., Yuan T., Tschannen M., Sun Z., Jacob H., Du M., Liang M., Dittmar R.L., Liu Y., Liang M. (2013). Characterization of human plasma-derived exosomal RNAs by deep sequencing. BMC Genom..

[167] Im H., Shao H., Park Y.I., Peterson V.M., Castro C.M., Weissleder R., Lee H. (2014). Label-free detection and molecular profiling of exosomes with a nano-plasmonic sensor. Nat. Biotechnol..

[168] Ambros V., Bartel B., Bartel D.P., Burge C.B., Carrington J.C., Chen X., Dreyfuss G., Eddy S.R., Griffiths-Jones S., Marshall M. (2003). A uniform system for microRNA annotation. RNA.

[169] Wahid F., Shehzad A., Khan T., Kim Y.Y. (2010). MicroRNAs: Synthesis, mechanism, function, and recent clinical trials. Biochim. Biophys. Acta.

[170] Fang S., Lee H.J., Wark A.W., Corn R.M. (2006). Attomole microarray detection of microRNAs by nanoparticle-amplified SPR imaging measurements of surface polyadenylation reactions. J. Am. Chem. Soc..

[171] Zhang D., Yan Y., Cheng W., Zhang W., Li Y., Ju H., Ding S. (2013). Streptavidin-enhanced surface plasmon resonance biosensor for highly sensitive and specific detection of microRNA. Microchim. Acta.

[172] Ding X., Yan Y., Li S., Zhang Y., Cheng W., Cheng Q., Ding S. (2015). Surface plasmon resonance biosensor for highly sensitive detection of microRNA based on DNA super-sandwich assemblies and streptavidin signal amplification. Anal. Chim. Acta.

[173] Hao K., He Y., Lu H., Pu S., Zhang Y., Dong H., Zhang X. (2017). High-sensitive surface plasmon resonance microRNA biosensor based on streptavidin functionalized gold nanorods-assisted signal amplification. Anal. Chim. Acta.

[174] Rycenga M., Cobley C.M., Zeng J., Li W., Moran C.H., Zhang Q., Qin D., Xia Y. (2011). Controlling the Synthesis and Assembly of Silver Nanostructures for Plasmonic Applications. Chem. Rev..

[175] Joshi G.K., Deitz-McElyea S., Johnson M., Mali S., Korc M., Sardar R. (2014). Highly specific plasmonic biosensors for ultrasensitive microRNA detection in plasma from pancreatic cancer patients. Nano Lett..

[176] Lee J., Park J., Lee J.Y., Yeo J.S. (2015). Contact Transfer Printing of Side Edge Prefunctionalized Nanoplasmonic Arrays for Flexible microRNA Biosensor. Adv. Sci..

[177] Guo L., Lin Y., Chen C., Qiu B., Lin Z., Chen G. (2016). Direct visualization of sub-femtomolar circulating microRNAs in serum based on the duplex-specific nuclease-amplified oriented assembly of gold nanoparticle dimers. Chem. Commun..

[178] Blackie E.J., Le Ru E.C., Etchegoin P.G. (2009). Single-molecule surface-enhanced Raman spectroscopy of nonresonant molecules. J. Am. Chem. Soc..

[179] Driskell J.D., Seto A.G., Jones L.P., Jokela S., Dluhy R.A., Zhao Y.P., Tripp R.A. (2008). Rapid microRNA (miRNA) detection and classification via surface-enhanced Raman spectroscopy (SERS). Biosens. Bioelectron..

[180] Guven B., Dudak F.C., Boyaci I.H., Tamer U., Ozsoz M. (2014). SERS-based direct and sandwich assay methods for mir-21 detection. Analyst.

